# Canes Build Ministers: Discipline, Memory, and Political Socialization at Thailand’s Premier Boys’ School, 1934–1942

**DOI:** 10.1080/14672715.2025.2502623

**Published:** 2025-05-16

**Authors:** Daniel Whitehouse

**Keywords:** Thailand, elite schooling, political memory, violence, ideology

## Abstract

This article examines how Suankularb Wittayalai—a Bangkok secondary school long associated with elite formation in Thailand—shaped the political outlook of its influential 1934–1941 cohort. Educated amidst the country’s fraught transition from royal absolutism to military nationalism, these students were subject to a regime of intensified discipline and ideological messaging. Yet this transformation did not emerge in isolation. The article shows how earlier traditions—rituals of hierarchy, codes of loyalty, and an ethos of national service—were preserved and redirected to support a more militant and ideologically ambitious state. Drawing on alumni memoirs and archival sources, the article explores daily life at the school from 1934 to 1941 and traces how these formative experiences were later mobilized in divergent ways: to legitimize authoritarian rule, inspire revolutionary struggle, or anchor a politics of virtue. By following the long emotional afterlives of a single cohort, the article shows that elite schooling generates not a single political trajectory, but a repertoire of attachments and dispositions that remain active, contested, and politically generative long after students leave their classrooms.

## Introduction

The museum at Suankularb Wittayalai secondary school contains a 1930s classroom carefully staged with authentic materials. Timeworn wooden desks are etched with adolescent graffiti; a dusty chalkboard poses an unfinished math problem; and on the teacher’s lectern, a school register lies open, its inked names slowly fading into yellowed paper. At the center of the display hangs a sepia portrait. It shows a lean, unsmiling man with Eurasian features and a clipped moustache. Beneath him stands a reliquary—a gilded pedestal draped in gold silk and softly lit. Resting across the silk are four polished bamboo canes. A placard reads:

Luang Seksunthorn, Deputy Headmaster, 1934–1941

Canes Build Ministers (ไม้เรียวสร้างรัฐมนตรี)

Alumni from this era achieved remarkable success due to corporal punishment. Reflecting on their formative years, alumni harbor no resentment toward their teachers. Instead, they offer effusive praise and contemplate their good fortune in receiving character training that steered them from wrongdoing toward the path of righteousness.

Without further elaboration, the canes are left to speak for themselves. Yet the story they evoke is far more complex. Among the generation of students who passed through Suankularb in the years following the fall of royal absolutism, these implements of discipline are remembered with an intensity that did not diminish with time. For some, they signified formative exposure to virtue and order. For others, they evoked fear, resentment, and enduring harm.

This article explores how Suankularb’s disciplinary regime was entangled in the ideological transformations of 1930s Siam. Focusing on the 1934–1941 student cohort—educated during the country’s volatile transition from absolute monarchy to military rule—it reconstructs how authoritarian pedagogy, physical punishment, and political socialization converged within the school, and how these experiences were later mobilized in strikingly different ways, justifying political projects ranging from revolutionary resistance to royalist consolidation. Rather than trace a single political legacy, I approach Suankularb as an emotional institution that cultivated loyalty and discipline, but also produced deep ambivalence. Many of the boys who endured Seksunthorn’s cane went on to become architects of postwar Thailand, as prime ministers, revolutionaries, state security chiefs, and communist partisans. Through close readings of personal memoirs and institutional archives, I offer a new perspective on how formative attachments to elite schools shaped the political outlooks, affiliations, and emotional lives of a generation that would define Thai politics for decades to come.

## The Suankularb cohort of 1934 –1941

The Suankularb cohort of 1934 to 1941 produced two prime ministers: Thanin Kraivichien (class of 1941), later regarded as modern Thailand’s most repressive leader, and Prem Tinsulanonda (class of 1938), a senior statesman whose long career spanned roles as army commander-in-chief (1978–1982), prime minister (1980–1988), president of the Privy Council (1998–2019), and regent (2016). Both men worked closely with former classmates. In his brief administration, Thanin appointed two of his 1941 peers—Suthi Natworathat and Pinyo Sathorn—as ministers of commerce and education, respectively.^1^ A third classmate, Air Marshal Sak Tharichatra, described by Thanin as a “beloved schoolfriend” who “truly helped,”^2^ declined a cabinet post but remained a trusted figure behind the scenes. Sak established a School for Psychological Warfare within the Air Force’s Department for Military Operations and handled “many things for national security … some that can be revealed, and some that cannot.”^3^

Prem drew on the loyalty of fellow graduates to an even greater extent. From his 1938 graduating class he appointed General San Chitrapatima as deputy commander-in-chief of the Thai Army (1980); ACM Siddhi Savetsila as foreign minister (1980-1988) and deputy prime minister (1986); Suli Mahasantana and Major General Sudsai Hasadin as minister attached to the prime minister’s office (1981-1988 and 1981, respectively); and Police Lieutenant Charn Manutham as public relations minister, deputy minister for transport and communications, and, later, minister attached to the prime minister’s office (1981-1986). Friends from adjacent grades also secured strategic positions. Admiral Sonthi Bunyachai (class of 1935) served as deputy prime minister from 1983 until 1988; Admiral Amorn Sirikaya (class of 1937), as deputy minister of transport and communications from 1983 to 1986; General Serm na Nakorn (class of 1939) as deputy prime minister from 1980 until 1983 and supreme commander of the armed forces (1980-1); Suraphon Chulaphram (class of 1940) as police commander (1981-1982); and General Harn Leenanont (class of 1941) as minister of agriculture and cooperatives from 1986 to 1988.

Prem’s relationship with his Suankularb brethren appears to have been mutually reinforcing: their loyalty bolstered his authority, and he rewarded it with access to resources. Within a week of being appointed prime minister, and while still serving as president of the Suankularb Alumni Association (a position he held from 1978 to 1982), Prem initiated the construction of a grand alumni headquarters, built on land allocated by the Crown Property Bureau close to his home.^4^ Intended as a monument to alumni prestige, the project ballooned beyond its fourteen million baht budget under the direction of Pridi Hiranpruet, club secretary and Prem’s undersecretary of foreign affairs. The headquarters was officially opened in March 1982, when Prem and Deputy Prime Minister Serm ceremonially declared it a space for the consolidation and celebration of the Suankularb brotherhood.^5^ In 1987, the Suankularb campus was designated a national heritage site and its 105th anniversary was broadcasted nationwide, complete with celebrity performances and appearances by senior political figures.^6^

Proximity to Prem’s Suankularb cohort conferred significant socio-political prestige. From 1979, soon after Prem became commander-in-chief of the Thai Army, alumni began submitting anonymous letters to the society columns of popular magazines, requesting that they publish lists naming his school peers now working in the police and armed forces, civil service, and medical professions.^7^ These lists functioned as discreet signals of elite affiliation, allowing alumni to subtly signal their connection to Prem’s influential inner circle. For Prem, the widespread presence of Suankularb alumni in state institutions offered a powerful mechanism for mobilizing his influence. Senior figures such as Siddhi Savetsila and Sudsai Hasadin, alongside younger alumni like Surayuth Chulanont (class of 1961) and Kramol Thongthammachart (class of 1953), formed a strategic sub-network within what Duncan McCargo has termed a “network monarchy,” the patronage system through which Prem advanced trusted allies and consolidated royal authority during the late Bhumibol era.^8^

Yet the 1934–1941 cohort to which Prem belonged did not speak with a single political voice. During Thailand’s ideological polarization of the 1960s and 1970s, former schoolmates surfaced as prominent leaders on opposites sides of Thailand’s Cold War divide, some as senior cadres of the Communist Party of Thailand (CPT), others directing the state’s campaign to destroy them. On the left, schoolfriends Ruam Wongphan, Charoen Wangnam, and Somkhwam Phichaikul (all from the class of 1941) developed a close-knit bond shaped by a shared rural upbringing and a mutual sense of exclusion from privileged peers like Thanin Kraivichien, whose daily arrival by car symbolized the social distance between them.^9^ Somkhwam resented the school’s nationalistic rituals, later describing being forced to “stand before the flagpole and chant, ‘everyone must love the nation, religion, king, and constitutional system.’”^10^ After graduation, Charoen joined the underground resistance against Japanese occupation and, by 1947, all three schoolfriends were CPT members, with Ruam and Somkhwam leading the Thai Youth Organisation, the nucleus of Thailand’s first student movement.^11^ Their ascent within the PCT was rapid. Ruam was appointed to the politburo; Somkhwam sat on the central committee; and Charoen, known by his *nom de guerre*, Comrade Samanan, served as deputy chairman (from 1955 to 1961) and secretary general (from 1961 to 1979). The trajectory of the communist movement shifted on April 24, 1962, when Ruam was arrested and became the first Thai citizen executed for communist activity. Charoen responded by initiating the *Paa Nam Baan* strategy, which relocated the party’s base to the rural uplands of Phu Phan and embedded operatives in remote villages to manage and reorganize daily life.^12^ He invoked Ruam’s final declaration—“Long live the Thai people”—as a rallying cry for the movement.^13^ Full-scale armed insurgency broke out in 1965. Somkhwam was imprisoned from 1967 to 1972; Charoen was wounded in combat in 1968. By 1969, state authorities had designated thirty-five of Thailand’s seventy-one provinces as “communist-infested sensitive areas”—a stark reflection of the party’s reach and the perceived scale of the threat it posed.^14^

A coordinated counteroffensive emerged in 1975, with key leaders drawn from the same Suankularb cohort. Supreme Court judge Thanin Kraivichien (class of 1941) acted as spokesman for *Nawaphon*, an ultranationalist organization of urban professionals. In televised speeches, Thanin framed progressive movements as existential threats to the monarchy and national order.^15^ Working in tandem with *Nawaphon,* Colonel Sudsai Hasadin (class of 1938) founded and commanded the *Red Gaurs*, a paramilitary force comprising disaffected technical students and local enforcers who were trained to act as the regime’s shock troops. Under Sudsai’s direction, the *Red Gaurs* torched buildings, assaulted demonstrators, and executed political opponents, becoming the most feared instrument of state violence in the escalating conflict.^16^ The third arm of the campaign was the Border Patrol Police (BPP), commanded by Police General Suraphon Chulaphram (class of 1940), once known to his classmates as a soft-spoken student who readily shared his workbook. Decades later, Suraphon presided over a vast internal security apparatus, directing paramilitary operations and psychological campaigns, including the Village Scouts, a state-sponsored group of rural citizens that, by 1978, counted over 2.9 million members.^17^

Alongside *Nawaphon* and the *Red Gaurs*, the BPP played a central role in one of the most shocking acts of political violence in modern Thai history: the massacre of at least forty-one unarmed student protesters at Thammasat University on October 6, 1976.^18^ The attack was exceptionally brutal, with lynchings, sexual violence, and indiscriminate killings carried out in broad daylight. Appointed prime minister by King Rama IX in the wake of the massacre, Thanin launched an uncompromising crackdown on student activists, forcing thousands into the jungle to join the CPT.^19^ The political consequences of this period were profound. As David Morrell and Samudavanijap Chai-Anan later reflected, “rightist vigilantes and Border Patrol Policemen destroyed much of the country’s new generation of leaders, psychologically if not physically.”^20^ In 1980, Prem declared an amnesty for former insurgents. This marked the formal end of the conflict and paved the way for the CPT’s eventual disintegration. Out of this rupture, Prem contributed to the formation of network monarchy, positioning the crown as a stabilizing moral compass and final safeguard against renewed polarization.

In the Suankularb Museum, there is no reference to the cohort’s ideological divides, nor to the fact that its members led opposing sides in Thailand’s Cold War battlegrounds. Instead, the exhibit presents the 1934–1941 cohort as a cohesive bloc of *khon di* (good people) molded by the firm hand of Deputy Headmaster Luang Seksunthorn. This narrative gained institutional traction during the 1980s, as Prem and his classmates ascended to political prominence. “Suankularb was serious in teaching us to be *khon di*,” Prem later recalled, praising Seksunthorn’s severity as formative exposure to moral discipline. “He needed only clear his throat, and our hair stood on end.” ^21^ In commemorative publications, Prem portrayed the school as a moral crucible which, through strict discipline and routine, forged not just students but upright leaders.^22^ As prime minister, Prem drew on the language of *khon di* to legitimize his vision of state authority grounded in moral rectitude rather than electoral legitimacy. He advocated for governance by ethical appointees rather than politicians, often framing his own leadership as an extension of the values instilled at Suankularb. With many of his Suankularb peers occupying senior roles across the state, what began as a personal recollection swiftly calcified into institutional doctrine. Fellow alumni contributed their own retrospectives, collectively reimagining their alma mater as a cradle of civic virtue, with Seksunthorn elevated to the status of moral patriarch. These accounts often blended memory with veneration, as in a poem by Chalonglak Aksonsan (class of 1941):

Seksunthorn, a man of care, regulation, and discipline
corrected our manners for all to see
personal morality was his goal.
If insolent, he would chasten all evil
to build *khon di*.^23^

“Tears well in my eyes contemplating the kindness of my old teacher,” later wrote Sawat Thamikra (class of 1939), who went on to a career as a professor of engineering at Chulalongkorn University.^24^ “If he didn’t love us, why would he have hit us so?” By the time the Suankularb Museum opened in 2002, this interpretation had solidified. Seksunthorn’s canes were installed as artefacts for of institutional veneration.

But reverence was not unanimous. In 2009, Thanin Kraivichien—by then eighty-two years-old—broke ranks with his cohort’s prevailing nostalgia, arguing that Seksunthorn’s violence did not nurture morality but concealed unchecked rage. “If you were viciously hit as a child,” he wrote, “it may be that you are not moral—only that the beatings concealed the foul temperament (*arom rai*) of the castigator.” ^25^ He argued that the true legacy of Seksunthorn’s regime was not ethical integrity but lasting psychological harm: “A passionate fury (*khiaet khaen*) that only finds release by attacking others in turn.” For Thanin,the lesson was clear: “violence breeds violence.”^26^ It is a striking revelation, coming from a figure so closely associated with state brutality.

The debate over Seksunthorn’s legacy exposes a tension at the heart of Suankularb’s institutional memory. Understanding how this tension emerged requires looking back to the school’s origins, when it was first tasked with producing a new kind of subject for a centralising royal state.

## Suankularb under royal absolutism

Suankularb was established in 1881 amid a far-reaching reorganization of Siam’s political order. Seeking to curb the autonomy of hereditary officeholders and provincial elites, King Chulalongkorn (r. 1868-1910) pursued a centralized bureaucratic model that demanded new forms of training and credentialing. Skills previously marginal to elite formation, such as literacy, numeracy, and record-keeping, were suddenly reframed as core administrative skills.^27^ As commander of the dwindling Royal Pages, Prince Damrong Rajanubhab (King Chulalongkorn’s half-brother) saw his regiment as a promising foundation for training educated young nobles. At just eighteen, and with scant resources, he sought permission to convert the cramped and dilapidated Suankularb Palace on the northern fringe of the royal compound into a military school.^28^ Classes began in September 1881 with an initial cohort of ten lieutenants with aristocratic backgrounds. Academic affairs were supervised by Phraya Owatworakit Prian (1826-1906) a seasoned royal scribe who implemented a curriculum based on the *Munlabot Banphakit*, a series of rudimentary language exercises devised by his former colleague, Phraya Si Sunthorn (1822-1891), who served as pedagogic consultant. Luang Surayuthyorthahan (1857-1903) enforced military discipline, ensuring recruits wore the regulation blue-black uniform, attended classes, participated in physical drills, carried firearms, and slept on-site.^29^

A year later, rising demand quickly outstripped the regiment’s limited capacity, prompting major reform. In consultation with King Chulalongkorn, Damrong opened the school to the sons of lower-ranking nobility. This shift supported the King’s broader ambition to create a network of royal schools that would supply the expanding bureaucracy with competent officials. In its civilian iteration, Suankularb offered a template for these ambitions while addressing the king’s growing anxieties about the moral decline of young nobles, whom he accused of “roguishness” (*prarit sephen*) and “thuggery” (*pen nakleng huamai*). He tasked the school with serving as “a channel through which they might know right and wrong.”^30^

Discipline lay at the heart of Chulalongkorn’s moral reform agenda, but applying it to aristocratic boys proved politically delicate. Many noble families remained suspicious of secular schooling and deeply uncomfortable with the prospect of their sons being reprimanded by social inferiors. A comparable challenge had surfaced six years earlier at a palace school led by British educator Francis George Patterson, whose modest use of corporal punishment—he once slapped two students’ palms with a wooden ruler—alienated parents. ^31^ Although the *Royal Gazette* had issued public assurances that instructors were “given no dispensation to scold, strike, or be impolite to students,”^32^ the school lost its fifty pupils and quietly folded when Patterson’s contract expired after eighteen months.^33^ This preference for restrained discipline reflected longstanding norms within elite-favored monasteries, where aristocratic boys had traditionally received their education. Si Sunthorn, a respected monk and adviser to Suankularb, discouraged physical punishment for high-born pupils, warning that they grew “weary and dispirited” under harsh language or force. ^34^ He accepted that “rough punishment” might be appropriate for peasant children but urged that all students be treated with courtesy (*atthayasai*), and that discipline avoid distress or humiliation. His contemporary, the monk Phae, likewise called for “compassionate mercy” as the guiding ethos in education.^35^ Damrong—the final student enrolled at Patterson’s failed school—was acutely aware of these sensitivities. At Suankularb, he introduced a similarly light-touch ethos, relying on free uniforms and daily stipends to encourage attendance.^36^ The approach helped retain students and expand the school, albeit at the expense of inconsistent discipline.. Prince Bidyalongkorn (1877-1945), who enrolled in 1886, recalled being regularly beaten by senior students, and admitted to “torturing” those beneath him.^37^

The turning point came in 1892, when admission was extended to high-achieving commoners, expanding the student body to over three hundred and straining the spatial limits of the royal compound. In response, the school was divided and relocated. A smaller English-language section remained within the palace grounds, while the larger Thai division moved to four converted outbuildings on the grounds of Wat Mahathat.^38^ It was there, in 1898, that Suankularb implemented its first system of student conduct, endorsed by Prince Kitiyakara Voralaksana (1874-1931), director-general of the Department of Education and a former pupil. The code identified twelve primary infractions, including fighting, smoking, cursing, and wandering, and established a five-tier system of penalties ranging from detention to expulsion.^39^ The code upheld the prohibition on corporal punishment. The following year, a parallel set of protocols was introduced for faculty, aimed at reducing absenteeism and tightening oversight.^40^ Together, these reforms signalled a clear departure from a personalized, deferential model of elite instruction, laying the foundation for a more standardized system that enabled future institutional consolidation.

By 1909, Suankularb had become the kingdom’s premier educational institution, widely esteemed for its academic rigor. Under the guidance of alumnus and Minister of Education Pia Malakul (1867-1917), a protégé of Damrong , plans were initiated to unify its divided sections on an expansive new site on Phahurat Road, in a manner befitting its national stature. Pia, a former ambassador to the United Kingdom, dreamed of a school beyond anything Siam had ever seen: “a great seat of learning that in other countries is called a college … or some other word for which the Thai do not yet have a name.” ^41^ When completed after two years, the school was the longest building in the kingdom, a 193-meter neoclassical structure with 166 windows, 164 doors, and 37 expansive rooms arranged along a grand colonnade, bordered by manicured lawns. A separate library, housed in the similarly ornate Teacher’s Association building, provided space for faculty exchange and research. Suankularb became a showcase for visiting dignitaries observing the kingdom’s pedagogical aspirations.^42^

The driving force behind Suankularb’s expansive transformation was a cadre of British headmasters, supported by Thai administrators with strong anglophile leanings. In 1894, the Education Department hired twenty-four-year-old Englishman Ernest Young to direct Suankularb’s new English school. A working-class graduate of Borough Road College, Britain’s first teacher training institution, Young typified a rising class of professional educators. Disillusioned with Oxbridge educated tutors, the Ministry of Religious Affairs (which oversaw education in Siam) turned to Young’s practical training and administrative skill to shape the emerging institutional framework of the new school.^43^ Though his tenure was brief (he returned to England in 1898) Young’s appointment set a lasting precedent. Over the next thirty years, four additional Borough Road graduates served as headmasters, each working to emulate the culture of their alma mater and act on Young’s critique that Suankularb lacked “esprit de corps.”^44^ These men consolidated Suankularb’s institutional identity around three core pillars: its adoption of a stricter disciplinary framework; its function as a feeder for the civil service; and its cultivation of loyal alumni. By reconfiguring the school’s internal culture, these reforms laid the emotional and institutional foundations that the 1934–1941 cohort would inherit, shaping how they experienced discipline, internalized hierarchy, and attached moral value to state service.

Departing from the conventions of their British public school contemporaries, these early Borough Road graduates, shaped by a progressive model of teacher training, promoted a disciplinary ethos based on routine, consistency, and behaviorial modelling.^45^ William Johnson (1895–1897), Young’s successor, maintained the Palace School’s ethos of gentle discipline by forgoing corporal punishment in favor of a system that rewarded consistent attendance with gold, silver, and bronze medals.^46^ Top-performing students were awarded the exclusive privilege of sitting at the “golden desk,” an elaborately carved seat prominently positioned at the front of each classroom.

The first documented use of corporal punishment during the Borough Road era came in 1910, under Headmaster Henry Spivey (1903–1915), when several students were caned for skipping school to play football in the Dusit area, a common form of truancy known as a “strike.”^47^ But physical punishment remained rare and undertaken with reluctance. When Insri Chantarothit (later the minister of agriculture and cooperatives) was caught hiding in the Teacher’s Association library in 1914, he recalled that the *farang* teacher who caned him expressed visible hesitation.^48^ Tardiness was typically met with scolding rather than corporal punishment, as few households owned timepieces, and many pupils travelled long distances to class. In the final months of Spivey’s tenure, the newly arrived A.G. Beaumont, a known taskmaster, disciplined latecomers by parading them to the front of the classroom “with sheepish faces,” all the while recounting his own punishing commute by boat and tram.^49^

The death of Henry Spivey in 1915 marked a turning point in Suankularb’s disciplinary culture. His successor, Norman Sutton, ushered in a more assertive, emotionally charged phase in the school’s governance. Of the British headmasters, Sutton was remembered as both the most exacting and the most admired. His defining dictum, delivered in a thick Yorkshire accent, captured the hierarchy he expected in the classroom: “I am the teacher, and you are my student. You must obey me and act under my authority. If not, we cannot be teacher and student.”^50^ He famously caned an entire class that refused to complete a difficult test, insisting between blows that students accept whatever truths he declared. “If I say one plus one is three, you believe it. If I tell you the Nile is in China, you believe it. Remember this and do not disobey me again.”^51^ New students were warned—“do anything wrong, and Sutton shouts, ‘bend down’ and canes you.”^52^ This severity was partly theatrical. Among his colleagues, Sutton derided corporal punishment as the crutch of a “second-rate” teacher, and many students saw him as more protective than punitive.^53^ In 1931, just months after his return to England, sixteen Suankularb students staged an early morning raid on a girls’ Christian school and became the focus of a ministerial inquiry. Among them was Pradit Phanichkan, who later became the director-general of public prosecution. Years later, Pradit reflected, “Mr. Sutton would have protected us,” a remark that underscores Sutton’s role as a mediator who shielded his students from the more punitive dimensions of state authority.^54^ Sutton’s model of rule—strict but affectively charged—set the emotional and disciplinary tone that would later be intensified a few years later under Seksunthorn.

By the late 1890s, the Borough Road headmasters had begun to remake Suankularb, not only by formalizing its disciplinary structures, but by nurturing a powerful emotional affiliation with state service, casting the school as the most prestigious entry point into Siam’s expanding modern bureaucracy. In collaboration with Pia Malakul, Headmaster Johnson devised a demanding three-day examination to identify “white elephants,” exceptional students who would receive royal scholarships to study overseas, an opportunity previously confined to the hereditary elite.^55^ The exam, modeled on Britain’s Queen’s Scholarship (which Johnson had won as a schoolboy), closely aligned with Suankularb’s English-language curriculum, giving its students a clear advantage. Suankularb students soon dominated the lists. Between 1897 and 1911, the school produced twenty-four King’s Scholars and forty-one additional state-funded scholars for overseas study. ^56^ In 1912, its pupils secured nine of ten royal awards; in 1931, they swept the board.^57^ This success brought prestige to both graduates (who often secured exceptional ministerial salaries)^58^ and the school. Admission became highly competitive. Some students repeated their final year multiple times to re-sit the arduous scholarship exam. ^59^ Headmasters marked these achievements with symbolic pageantry such as parades, honor boards, and farewells, imbuing state service with emotional weight and presenting it as a fulfilment of Suankularb’s deeper institutional purpose. “We were the white elephants come in from the forest,” recalled Pradit Phanichkan. “We went forth to benefit the entire country, bearing a prestige that travelled far and wide.”^60^

Long before the 1934–1941 cohort leveraged their school ties for political advantage, Suankularb’s British headmasters laid the emotional groundwork for such emotional bonds. As they consolidated the school’s status as a bureaucratic feeder school, they launched a parallel campaign to instil institutional loyalty through sports and emerging forms of school-based affiliation. Following Johnson’s elevation to chief inspector of schools in 1897,^61^ Headmaster Ernest Smith (1897–1903), a former Aston Villa XI player, introduced physical training and launched Siam’s first inter-school football tournament.^62^ Drawing on their Borough Road experiences and supported by generous state funding, Smith and his successors built what became the most advanced sporting infrastructure in the country.^63^ By 1913, Suankularb boasted Siam’s first gymnasium, international-standard pitch, and permanent boxing ring.^64^ Matches attracted large crowds and encouraged intense inter-school rivalries. Royal patronage from King Vajiravudh (r. 1910-1925), who presided over the Suankularb Sports Club, lent these events further symbolic weight, setting up Suankularb as a national stage for the performance of his Edwardian-inflected ideals of royalist masculinity.^65^ By the tenure of Headmaster Norman Sutton (1915–1930), who had been Borough Road’s most celebrated all-round athlete,^66^ this vision of Suankularb had become fixed in the national imagination, as affirmed by *The Bangkok Times*:

The youth of Bangkok have stronger, larger, and more resilient bodies these twenty years past. These days, a sporting fixture with a *farang* squad will likely result in a Thai victory. This is a far cry from the past when the small and feeble stature of the local population assured perpetual defeat. Be in no doubt about it, this evolution of physicality and psyche is the result of the various sporting exercises spearheaded by Englishman and teacher, Mr Norman Sutton.^67^

Suankularb’s embrace of athleticism soon merged into a broader performance of martial nationalism. Beginning in 1912, the school’s playing fields hosted the annual jamboree of the *luk suea* (Siamese Scouts), part of King Vajiravudh’s efforts to mold patriotic youth in the image of Robert Baden-Powell’s British scouts as a counter to rising anti-monarchist sentiment. ^68^ Activities included canal swimming, tree climbing, and fire-making, culminating in elaborate war games and royalist parades held on the school’s grounds each July. Suankularb’s own scouts occupied leading roles, not only during the jamboree, but in public life more broadly. In December 1927, the troop extinguished a large house fire on Charoen Krung Road.^69^ A photograph from the day shows teenage boys in uniform, some barely fifteen, directing water at a smoldering façade—an early expression of the embodied discipline and patriotic performance that would intensify at the school in the 1930s.

By 1930, the symbolic architecture that would bind the 1934–1941 cohort was firmly in place. Drawing on the traditions of his alma mater, Sutton introduced a school insignia, motto, song, and sporting colors—external markers of unity that gave form to an imagined institutional fraternity. He also established *Suankularb Withaya*, a school magazine that offered pupils a script for communal memory, including updates from past cohorts, reminiscences from distant provinces, and reverent profiles of successful alumni. In the regular column “School News and Old Students,” pupils were invited to feel part of an unbroken lineage. When alumnus Second Lieutenant Aat Sucharitkul died during a training exercise at RAF Hawkinge in 1931, the magazine urged students to gather at Hua Lamphong station to receive his ashes. “Even though he has passed,” the editors wrote, “we will never forget him and honor his memory always.”^70^ Such affective bonds had no precedent among the earlier Palace School cohorts, who had not been socialized into a culture of sentimental affiliation. When, in one of his final acts as headmaster, Sutton attempted to formalize these bonds through the establishment of a Suankularb Alumni Association, he was met with indifference from senior alumni who viewed the expanded student body as a dilution of their elite identity.^71^ Their refusal foreshadowed the rupture of 1932, which would define the contested political terrain in which the 1934-1941 cohort was socialized.

## Suankularb after the fall of absolutism

On the morning of June 24, 1932, a fifteen-year-old Suankularb student named Prakep Khlongtruat left his home on Soi Set Siri and cycled south along Rama V Road. Rehearsing English vocabulary for his first-period class, he noticed something strange—the streets were empty. “Something wasn’t right,” Prakep later recalled in his memoirs, written nearly forty years after the event. As he neared the looming façade of the Ananta Samakhom Throne Hall, he saw armed soldiers encircling the plaza with rifles and machine guns. A naval officer waved his gun at him. “Student, go quickly or you’ll catch a stray bullet,” he shouted. Moments later, a tank barrelled past. Prakep immediately understood that the absolute monarchy was over.^72^

When Prakep arrived at the school gates, he found his schoolfriends gathered in excited huddles, listening for the distant rattle of machine guns. Around eleven a.m., confused staff and senior students were summoned to the main hall. A tank had entered the gates, blocking the road behind the school, and soldiers filled the grounds. One thrust a pamphlet into Prakep’s hand, while another climbed the stage, outlining in barked phrases the political objectives of the People’s Party, the group behind the unfolding coup. Then a civilian stepped forward and identified himself as Sanguan Tularak. He demanded Suankularb’s loyalty and instructed them to head straight to the Ananta Samakhom Throne Hall to declare their allegiance. Prakep remembered:

When Mr. Sanguan stopped speaking, the hall mumbled, unsure how to proceed after the volley of information. He then produced a black Browning pistol from his pocket and raised it in our direction as if to shoot, declaring, “If anyone disagrees with this march, say so and I will plug you straight away.” We all shouted, “we agree, we agree.” ^73^

The students complied, arriving at the Throne Hall with pre-printed placards reading “We Support the People’s Party.” They were welcomed by Captain Phibun Songkhram, one of the coup’s military leaders, who praised their demonstration of loyalty.

For the coup’s leadership, securing the visible support of Suankularb was both a tactical maneuver and a symbolic triumph. Just hours earlier, its alumni had stood on both sides of the unfolding confrontation. Pridi Banomyong, the ideological architect of the People’s Party, later traced his political awakening to a Suankularb classroom, where a fable about the despotic kingdom of Sawatthi first stirred his anti-absolutist convictions.^74^ It was Pridi who ordered Sanguan to mobilize the school’s students and faculty. Other prominent revolutionaries who were alumni included future Prime Minister Thawee Bunyaket (Suankularb Withayalai, class of 1918); Rear Admiral Luang Sangworathakit (class of 1917), who later served as deputy defense minister and director-general of customs; Luang Sinthusongkhramchai (class of 1914), who held multiple cabinet positions including, at various times, as minister of economics, education, agriculture, and interior, and also served as commander-in-chief of the Royal Thai Navy; and Lieutenant Commander Luang Suphachalasai (Suankularb English School, class of 1912), who later served as minister of the interior and of education. From the older generation came loyalists. Those arrested after the coup included the school’s founder Prince Damrong, detained just days after his seventieth birthday; Interior Minister Prince Paribatra Sukhumnadhu (Palace School, 1891); and Phraya Senasongkhram (English and Thai School, c.1903), who was shot while being apprehended. Minister for Commerce and Transport Prince Purachatra Jayakara (Suankularb Palace School, 1894) escaped via rail to the king’s summer residence at Hua Hin.^75^

Suankularb became a steady ally of the fledgling regime. While students at St. Gabriel’s and Assumption College staged public rallies demanding new rights, Suankularb’s pupils remained notably silent.^76^ In October 1933, many donned their scout uniforms to man prisons and ferry ammunition to the frontlines in support of the government’s efforts to suppress Prince Boworadet’s royalist countercoup.^77^ Some boys exchanged fire with rebel forces at Bang Sue and Laksi, comparing their experiences to scenes from *All Quiet on the Western Front*, recently screened at the Bang Krabue picture house. When the rebels surrendered after two weeks of fighting, the students returned in triumph, leading a victory parade through the northeastern city of Rachasima. Suankularb was awarded the newly introduced “Medal for the Safeguarding of the Constitution,” by Lieutenant-Colonel Phibun, who emerged from the victory emboldened and newly dominant.^78^

In the months that followed, Phibun sidelined the liberal wing of the People’s Party and moved decisively away from parliamentary pluralism. Drawing on fascist Europe and imperial Japan, he envisioned a militarized, expansionist state in which youth served as both subjects of indoctrination and agents of national renewal. Suankularb became a key laboratory for Phibun’s militarized nation-building. Each morning, Phibun’s grey Renault idled outside the school gates, delivering his son Prasong—a quiet gesture that signalled the school’s new role in his nation-building narrative.^79^ In 1934, he established the *Yuwachon* Corps, a youth organization patterned after the Hitler Youth, which fused military discipline with ideological training.^80^ Voluntary at other institutions, Suankularb imposed mandatory *Yuwachon* participation in 1936.^81^ Training was entrusted to Major General Pun Punyaruetseni, veteran of the Boworadet Rebellion,^82^ who repurposed the school’s playing fields for twice-weekly weapons training and physical drills.^83^ Students had to wear a mandatory *Yuwachon* uniform: a dark khaki shirt and shorts, paired with black shoes, knee socks, and a peaked military cap emblazoned with the red-band motto “Love Your Country Over Your Life*”* (*rak chat ying chip*). Each shirt bore a personal identification number, enabling the swift reporting of disciplinary infractions. A forgotten salute, unruly hair, or casual play fell under the purview of the military police, who could deny graduation. By 1939, this uniform had become regular attire.

Physical training was accompanied by regular ideological instruction. Each afternoon, members of the 1934–1941 cohort assembled for lectures delivered by Mr. Nop Palakawong Na Ayutthaya and Mr. Prasert Sapamut, Thai language teachers who extolled the military as the backbone of national power and the guarantor of Siam’s future.^84^ Deliberately stirring, these presentations provided students with some of their most formative political influences. Chinwut Sunthornsima, who would later become a general and serve as a cabinet minister under Prime Minister Chuan Leekpai (1992–1994), recalled being “spellbound with gratitude and patriotic feeling” during these sessions, describing himself as “ready to sacrifice everything for the nation and society … this feeling has stayed with me to this day.” ^85^ Efforts to cultivate martial fervor deepened the emotional charge of everyday peer interactions, which demonstrated a rising tolerance for physical confrontation. Fights broke out with increased regularity, sometimes encouraged by PE teacher Chalerm Thaiwong, whose loyalty to *Yuwachon* ideals manifested in organized gymnasium bouts, where disputing students were made to fight before assembled classmates.^86^ Occasionally, these conflicts spilled into the soot-streaked forecourt of the adjacent Wat Liap power station, where students set their own rules and appointed referees.^87^ These ad hoc contests distilled a competitive, self-policing culture in which domination was both sanctioned and internalized. “It was every man for himself,” later wrote Somkhwan Pichaikul, a future Central Committee member of the CPT and staunch critic of Phibun’s regime.^88^

The aggression honed on the gym floor was mirrored in a classroom pedagogy that prioritized ethnolinguistic purity over cosmopolitan ideals. In 1935, Suankularb abandoned the bilingual curriculum of the Borough Road instructors and declared Thai language proficiency essential, forcing students who failed the subject to repeat the entire academic year. ^89^ Two years later, Suankularb stopped hiring new British staff, bringing to a close its longstanding partnership with Borough Road College. Arthur Churchill, the final English headmaster, stayed on in a diminished role, joined only by Mr. Deagan and Mr. Silverthorn, whose decisions to remain increasingly marked them as relics of a vanishing era.^90^ A year later, in lockstep with government policy, Suankularb fired all staff of Chinese and Indian descent. The directive, framed in language that echoed the racial purity rhetoric of Nazi Germany, swept away the multiethnic texture of everyday school life.^91^ First to vanish was the noodle stall, its Sino-Thai proprietor departing without ceremony. The satay vendor left next, setting up shop in Thonburi under the nostalgic name “Suankularb,” (the shop continues to operate under this name). Then, the quiet disappearance of familiar figures: Uncle Jek, the caretaker in his pressed shirt, his wife who sold pens and notebooks, Mr. Rang, the half-blind Indian guard, and Mr. Si, the bean vendor known for his generosity and pride in working at Suankularb.^92^ These people had formed the everyday fabric of Suankularb’s communal life.

Steeped in *Yuwachon* rhetoric, the 1934–1941 cohort remained largely indifferent to these purges. They focused instead on hosting school fairs and charity drives to raise funds for Phibun’s military campaigns in Indochina, proudly presenting gifts, including firearms, to soldiers departing for the front. At one 1940 gathering, attendees paid to pelt tennis balls at a schoolmate dressed as Auguste Pavie, the former French consul synonymous with Siam’s loss of Laos in 1893. “Significant funds were raised, channeling pain and anger,” recalled alumnus Kamol Khemthong.^93^ The emotional pitch escalated days later at a rally in Sanam Luang, where chants of ancestral sacrifice filled the air. The cohort absorbed every message. “We were in the age of transforming Thailand into a superpower,” an anonymous alumnus later wrote. “We followed the trajectory of Germany, the great Western superpower. It was a very serious time.”^94^

On the morning of December 8, 1941, the illusion of Thai self-determination gave way to geopolitical reality. With barely a shot fired, Japanese forces entered Bangkok and the city fell under occupation. Years of martial rhetoric had primed the 1934–1941 cohort to receive the occupiers with curiosity rather than fear. They marvelled at the discipline of the Japanese soldiers and the arms they carried.^95^ Allied bombing soon followed, and the boys enjoyed sprawling across the playing fields, identifying aircraft types as they targeted nearby sites. In 1945, a direct hit destroyed two classrooms at the eastern end of the Long Building, though no lives were lost. By then, Suankularb had already shuttered, with all students issued final year certificates in January 1942. Historians would remember them as “Tojo’s class”—a cohort fast-tracked into adulthood by foreign occupation. ^96^

The institutional transformations at Suankularb following the 1932 Revolution shaped a cohort whose experiences diverged markedly from those of earlier generations. Where previous students were educated in an environment grounded in royalist paternalism and bureaucratic preparation, the 1934–1941 cohort came of age in a school reconfigured by ideological urgency, militarized discipline, and heightened state control. Trained for heroic futures in a rising Thai state, the cohort graduated into occupation, postwar uncertainty, and a shifting international order. . In later life, alumni recalled their schooldays in visceral detail, with the figure of Deputy Headmaster Luang Seksunthorn looming largest in their accounts.

## Life under Luang Seksunthorn

Dressed in a pristine white Raj-style jacket and blue *chong kraben,*^97^ Seksunthorn stationed himself at the school gate at seven each morning and lit a cigarette. He favored the *Saphan Pho* blend, an acrid tobacco popular among the local toughs who loitered at the Sampheng market.^98^ As the first student passed through the gates, Seksunthorn began his inspection, with hair his most pressing concern. In 1934, the Education Ministry stipulated that male students’ hair must not exceed four centimeters in length. Seksunthorn enforced a stricter limit of two, measuring with a bamboo cane notched to the millimetre to ensure compliance.^99^ Students who exceeded this limit received six strokes of his cane, administered immediately. An alumnus described this procedure:

[Seksunthorn] ordered the boy to bend forward and lifted his shorts to expose the skin at the back of the legs … he raised the cane as high and far back as his hands could extend then brought it down with all his might, ensuring each stroke did not merely glance off, but scourged the area with bleeding welts. After the first strike, Seksunthorn began to lecture the student, pausing for the second, third, fourth strike and so on until the punishment was concluded.^100^

Clothing came next. Shorts had to end exactly six centimeters above the knee, with pleats strictly forbidden. Any infractions resulted in six strokes. A student who failed all checks could receive eighteen strokes before the school day had begun**.** ^101^

At eight o’clock, Seksunthorn withdrew from the gate and concealed himself behind a vestibule pillar, poised to surprise latecomers. Tardiness earned twelve strokes, with few exceptions entertained. One morning, a boy arrived dishevelled and limping, explaining that his tram had jumped the tracks, sending his carriage skidding down Charoen Krung Road. Seksunthorn issued thirteen strokes, the last an extra chastisement for failing to catch an earlier carriage.^102^ On another occasion, when Seksunthorn himself arrived late, he immediately fetched a cane from his office and beat his own son Maitri, with whom he shared his daily commute.^103^ Only once did he show clemency: a boy who arrived late with a flat bicycle tire was permitted to crawl to his desk in lieu of a caning. The boy never understood why:

I clasped my hands and performed a deep *wai* to express my gratitude … I set out an hour earlier every day after that. If history were to repeat itself, I knew that I would not receive the same response … Luang Sek’s regulations were divine law.^104^

The rest of Seksunthorn’s day was divided between teaching, administrative duties, and campus patrols during which he remained vigilant for infractions. As in all areas of his authority, punishment was administered with consistency: infractions such as fighting, teasing, walking on the grass, or loitering typically earned between six and twelve strokes.^105^ Merely witnessing a transgression could be grounds for punishment. Niphon Punyaphukna, later a major general, recalled a minor classroom dispute in which a coin, flung in anger, struck a bystander. Seksunthorn entered moments later, dismissed the students’ explanations, and caned the entire class in sequence, leaving evenly spaced welts along their thighs.^106^ Seksunthorn was particularly harsh with actions that risked tarnishing the school’s public reputation. Boys who leaned out of open windows and were visible to passersby on busy Phahurat Road, including young women enroute to the textile market, would receive a rapid succession of strikes that left bruises across their torsos.^107^

Still more egregious were attempts to communicate with female students from the neighboring Rajini girls’ school, including via paper airplanes.^108^ But leaving the school grounds provoked Seksunthorn’s greatest outrage. On one occasion, he chased a group of boys he caught climbing the fence, found them cowering at the back of an ice shop, and delivered their punishment in full view of the street, warning them that expulsion would follow any second infraction.^109^ Senior boys were often caned during Saturday school assembly as a spectacle to reinforce obedience among younger students.^110^

Seksunthorn expanded the scope of corporal punishment to also encompass athletic conduct and academic achievement. He personally reviewed examination scores and imposed one stroke with his cane for results under fifty percent and two strokes for those below forty. After major tests, he might enter a classroom with a list of thirty names, dispensing punishment in turn, although he often preferred to delay the act, letting anticipation ferment into dread. This policy proved brutally effective. “None of us studied out of innate diligence,” recalled one alumnus, “but out of fear of sinning.”^111^ Games, once synonymous with Suankularb’s *esprit de corps*, became an extension of Seksunthorn’s punitive authority. During football practice, he would stand at the center of the field and pick a boy to sprint the length. He then pursued him, thrashing his cane through the air, landing blows if he managed to approach.^112^ With caning the default disciplinary response, few students escaped unscathed. When Mr. Sawat, a soft-spoken English teacher, asked his class who had been beaten by Seksunthorn in the past month, every student raised their hand.^113^ In time, students developed a mordant shorthand for their new reality: *sai ti leum ti tok ti*—“late: caned; forget: caned; fail: caned.”^114^ To mitigate the pain of inevitable strikes, resourceful students began to reinforce the lining of their shorts with extra layers of fabric. Observing this, Seksunthorn adjusted his aim to more vulnerable areas where no protection could be applied, such as hamstrings or the back of knees.^115^

In those years, Suankularb was reputed to be the strictest school in the country. Corporal punishment was no longer a disciplinary tool but a structuring element of daily life.^116^ The crack of bamboo cane against flesh became an ambient feature of the institution’s everyday soundscape. “His office was above our classroom,” one student recalled, “and every morning we heard the thwack, thwack. It was a disturbing sound. It entered your soul.”^117^ The effect of this disciplinary saturation was cumulative. “School could be torment … there were so many rules. Far too many. And they were all absolute,” later wrote one alumnus.^118^ Seksunthorn encouraged his colleagues to adopt similar punitive methods. “Nearly all the teachers scolded,” remembered one student,^119^ a perception corroborated by others, who recalled that many teachers, including Mr. Foo, Mr. Chalerm, and Mr. Pian, routinely carried canes.^120^ In a Teacher’s Day ceremony following Seksunthorn’s death in 1941, Mr. Pian likened himself to a pot-maker, insisting he had to repeatedly beat his designs until they became “smooth and beautiful.”^121^ For many students, such metaphors did little to soften the impact. One alumnus recalled such intense dread that he occasionally lost control of his bladder.^122^ At Seksunthorn’s cremation ceremony at Wat Makut Kasatriyaram, a notched bamboo cane was placed in his casket, an unmistakable emblem of the disciplinary regime he had established.^123^

While Phibun’s educational reforms laid the structural groundwork for intensified discipline, it was Luang Seksunthorn’s personal authority that gave Suankularb’s regime its distinctive emotional tenor.. Born on March 10, 1894, near the auspicious Wat Saket in Bangkok, Luang Seksunthorn (originally named Louis Boch) was the first son of Lek, his Thai mother, and Herr E. Boch, a German émigré.^124^ Seksunthorn maintained a close though at times ambivalent relationship with his parents throughout his life, returning home punctually each evening to share dinner and assist with household duties. To colleagues, he acknowledged the emotional burden these commitments entailed. He confided that he felt “anger and depression" towards his parents which he was compelled to “hide away,” explaining, “they are old, and I fear they could not handle such things.”^125^ As a student, Seksunthorn was unremarkable. Eleven years at Assumption College yielded a level four French diploma but little else to distinguish him. Principal Spivey’s decision to hire him as a substitute French teacher in 1913 came with a condition: Boch was required to obtain an English teaching diploma, a stipulation that foreshadowed a career-long struggle for legitimacy among the credential-driven Borough Road staff.^126^

For two decades, Boch progressed slowly through Suankularb’s ranks, becoming a homeroom teacher and school band leader under Headmaster Sutton. His reputation divided opinion. He was admired for his loyalty to friends and indifference to rank but criticized for his volatility. In a culture of restraint, Seksunthorn stood out as impulsive and combative, “incautious to the point of hot temperedness,” a colleague later remarked. “If he thought to say something, he would say it immediately. If he wanted a quarrel, he would pursue it directly.”^127^ Seksunthorn’s teaching style was as unconventional as his temperament. Claiming to have invented a novel pedagogical method—essentially a heightened form of rote memorization—he boasted that he could teach any student to read French in five minutes. Those who failed were made to crawl on hands and knees to the back of the classroom. Such conduct frequently irritated Headmaster Sutton, who held him in low regard and impeded his advancement. Arthur Churchill, governing under less favorable political conditions, offered neither endorsement nor opposition. Though disturbed by the intensity of Seksunthorn’s punishments, he conceded its utility, remarking that “no teacher achieves silence like Seksunthorn,” and that such discipline was essential if students were to “grow to fear the law and the state.”^128^

Churchill’s diminished authority following the 1932revolution enabled Seksunthorn to consolidate his influence within the school. In 1936 the government promoted him to the rank of *Rong Amat Ek* and bestowed him with the honorific “Luang Seksunthorn” (pleasant enchantments). His salary rose eightfold, to 240 baht per month.^129^ Yet controversy continued to plague him. Alumnus Luang Bunpalitwichasat, who succeeded Churchill as headmaster in 1938, frequently expressed his anxieties that Seksunthorn’s punitive regime risked sparking a student rebellion. He was particularly concerned by a newspaper report from the eastern province of Chonburi, where a student had hurled his schoolmaster from a classroom window following months of ill treatment.^130^ Other colleagues expressed confusion over Seksunthorn’s insistence that students posed a hidden threat, finding his fixation on potential violence difficult to comprehend. One morning, Luang Samretwannakit, a respected alumnus-turned-teacher, encountered Seksunthorn pacing the school grounds and joked, “Claimed any victims today, Deputy Headmaster?” Seksunthorn responded without hesitation: “Turn your back on the tiger and you court death. It pounces and we fall. Then it vanishes back into the jungle, emboldened, ready to strike again.” Samretwannakit would later reflect: “I’ve known many teachers—more skilled and learned than I—but none whose pedagogy was as peculiar as Seksunthorn’s.”^131^

In 1936, even the increasingly assertive state sought to place limits on Seksunthorn’s radical approach. Education Minister Luang Sindhu Songkhramchai—one of Seksunthorn’s former students and a prominent revolutionary—introduced regulations requiring headmaster approval for all beatings by teachers. Seksunthorn predictably ignored the order and encouraged staff to proceed with caning as usual. When Minister Sindhu discovered Seksunthorn’s cane marks on his own son, he summoned his former teacher for an official rebuke. Seksunthorn, undeterred, turned the occasion into theater. He ordered students to form a ceremonial line as he departed the campus and, when he returned hours later, he unveiled a cigarette box he claimed was a gift from the grateful minister. “Let this be a lesson to you all,” he declared, “that parents entrust teachers as guardians of their children, granting us the right to scold and punish in the name of making good students.”^132^ Sindhu took no further action and Seksunthorn continued to wield unchecked disciplinary authority until his death five years later.^133^

For the 1934–1941 cohort, these formative experiences were neither easily forgotten nor easily processed. Reflecting back on his experiences in 1960, Samretwannakit remarked: “The name Luang Seksunthorn is firmly burrowed in the memories of surviving students.” Those memories were as complex and contradictory as the regime that produced them.

## The 1934-1941 cohort looks back

The men of Suankularb’s 1934–1941 cohort did not speak with one voice. In adulthood, they came to see their schooldays through widely divergent lenses. Some, like Prem Tinsulanonda, would later frame their education as a process of moral refinement, crediting it with producing *khon di* (good people) worthy of national leadership. Others, such as Thanin Kraivichien, viewed their experiences as a system of institutionalized abuse, condemning Seksunthorn’s disciplinary regime for nurturing cycles of fear and trauma. Still others, like Ruam Wongphan, left Suankularb determined to “resist fascist dictatorship” alongside friends who would go on to join the Communist Party of Thailand.^134^ What united them was not a shared political destination, but an intensity with which they remembered their schooldays.

Not all alumni processed their schooldays through the lens of ideology or national service. For some, the memory of Suankularb was intensely personal, entangled with grief, humiliation, and unresolved violence. Somchai Asonchinda, who transferred to St Gabriels in 1941 and later became a vocal critic of Phibun’s regime, offers one of the most haunting accounts. At sixteen, reeling from the sudden death of his father, he found himself repeatedly singled out for punishment by Seksunthorn, who labelled him “the scoundrel of the school” (*sian pracham rongrian*).^135^ Somchai recalled being “ashamed and furious” after a particularly brutal public caning for failing to wear his *Yuwachon* cap, that tore his trousers to shreds. The next morning, Somchai returned to school with his father’s chromium-plated revolver, intent on killing Seksunthorn. At the last minute, his friend Suphat intervened, urging Somchai to consider the disturbance such an act might bring to his father’s spirit. Somchai relented. But the memory lingered, and years later he learned that this classmate had taken a far darker path: in an act of extreme familial violence, Suphat murdered his wife and son with an axe before taking his own life. Reflecting on this tragedy, Somchai articulated what he saw as his cohort’s core psychological inheritance—a generation trained to conflate violence with care:

People say he was mad. I cannot believe this. I cannot believe my friend endured such misery that he was so determined to flee this world … Supat held his pride too dear. He was not psychotic. He killed because he loved, and he was fearful. He wanted the wretched life of his beloved wife and son to end with his … Supat killed because he loved. ^136^

As fathers, many of the cohort struggled to reconcile their school experiences with a changing society. In 1964, Prakep Khlongtruat, then forty-seven and head of a construction firm, received a call from Suankularb’s new headmaster, Phrong Songsengterm, an old classmate.^137^ The headmaster reported that Prakep’s teenage son had been skipping classes and underperforming, a pattern Songsengterm described as increasingly common.

“Why not cane them?” Prakep inquired, “that’s what Luang Seksunthorn did to us.” Songsengterm replied, “That era’s over, Kep. We’re in an age of democracy. Commit violence and you attract accusations of cruelty … These days parents call it barbaric.” Prakep didn’t argue. But as he set down the receiver, he found reassurance in the legacy of his cohort: “All of us,” he wrote later, “turned out commendable.” His thoughts turned to the former deputy headmaster., “I am really thinking about Luang Sek now … You caned them to scare them. If you want them to be *khon di*, you cannot be kind. The kids will mutiny.”^138^

Prakep decided to “honor the memory of Luang Sek and thrash the boy until [he] sees blood.” That evening, in the family garage, he bound his son’s wrists with rope and hoisted them over a beam until the boy hung, toes grazing the floor. With his wife and daughters watching, Prakep lowered the boy’s trousers and began to strike. “Dad,” his eldest daughter protested, “this is from the age of the African savage. We don’t hit anymore. Stop this.” He replied:

Good. If you don’t see savagery, how will you know modernity. Today I will let my children see the depth of a father’s love. If they are wrong, they must be punished. If you love your cow, you leash it. If you love your child, you strike it … We cannot spoil him, it will lead to his ruin. I have just started, there are eleven more to go till we reach the dozen.^139^

The scene became increasingly hysterical. Prakep’s wife threw herself over their son to absorb the blows, while his daughters, crying, attempted to coax their father outside for a walk. The distressed Prakep finally relented, leaving his wife to clean the boy’s wounds with antiseptic.

By the early 1960s, as Cold War anxieties were intensifying across Southeast Asia, former Suankularb students began to disagree publicly over the political implications of youth permissiveness. Speaking at a royal audience debate entitled “The Concerning Future of Youth” in January 1960, Wilas Maneewat (class of 1941) warned that leniency had made the younger generation “wild,” “insolent,” and “dangerous.” Wilas, whose father had gifted him a copy of *Mein Kampf* upon his admission to Suankularb, lamented, “In the past, adults threatened children; now children threaten adults. If we keep going, there will be nothing left.” ^140^ His views were challenged by Kukrit Pramoj (class of 1928), an alumnus of the comparatively gentler Sutton era, who dismissed Wilas’s assertion that severity cultivates virtue. “Adults whom children can bully are more childish than the children themselves,” he countered.^141^ Yet by the early 1970s, such liberal dissent was increasingly drowned out by the cohort’s intensifying ideological fracture. Two former classmates, Ruam Wongphan and Charoen Wangam, pursued radical paths of rural mobilization.^142^ Charoen led sustained efforts to agitate peasants, while Ruam, before his execution, reimagined the Communist Party of Thailand (CPT) as a nurturing “mother,” a symbol of care and collective resistance against entrenched power. ^143^ In stark contrast, their former classmate, Thanin Kraivichien, became a regular presence on national television during this period, denouncing student activists and communists as “un-Thai,” “traitors,” and “worms.”^144^ Thanin, rooting his political vision in the paternalism of ultra-royalism, elevated King Bhumibol to the status of a divine “father of the people,” a phrase he coined in 1976. ^145^ This paternal imagery was later institutionalized by Prime Minister Prem Tinsulanonda, who in 1980 designated the King’s birthday as Father’s Day. These rival visions spilled violently onto the streets. From 1965, Charoen presided over the CPT’s guerrilla war against the state, while by the mid-1970s, Sudsai Hasadin’s Red Gaurs were attacking young leftists in the cities.

By 1973, Suankularb became the focus of state surveillance, with unscheduled inspections by the Ministry of Education reflecting anxieties about the spread of radicalism.^146^ In 1974, a group of progressive students replaced the school yearbook with a 300-page polemic that denounced individual teachers and “the role of education in oppression and misdirection.”^147^

Conservative pupils, allegedly encouraged by some senior faculty, responded by assaulting the authors and burning the yearbook on the school field.^148^ The Thammasat University massacre of 1976 marked the apex of this violence, as state forces and paramilitary groups attacked student activists, leaving dozens dead. Yet within a few years, as the communist insurgency subsided, the 1934–1941 cohort was recast under Prem Tinsulanonda’s leadership. Rather than a site of fracture and bloodshed, Prem reimagined the cohort’s past as the foundation for a new vision of order: a network of *khon di* tasked with safeguarding the monarchy amid political uncertainty. That a single cohort could produce both insurgents and their suppressors, reformers and reactionaries, speaks to both the volatility of Thailand’s twentieth century and the institutional power of Suankularb during its most politicized years. Few generations have left such a contradictory and consequential legacy.

## Conclusion

In the Suankularb Museum, Seksunthorn’s canes remain enshrined beneath soft lighting, accompanied by a caption that simplifies rather than explains. For the 1934–1941 cohort, however, the meanings those canes carried were never stable. They evoked fear, admiration, humiliation, and pride, sometimes all at once. Suankularb functioned not just as a secondary school for Thailand’s elite male youth but also as a political institution, capable of impressing state ideology onto the moral and emotional worlds of its students. What it produced was not a shared worldview, but a cohort bound by deep and contradictory attachments.

The political significance of this generation lies not only in their divergent adult paths—as revolutionaries, reactionaries, ministers, and partisans—but also in the institutional and emotional conditions that made their fragmentation and later reconvergence possible. The post-1932 educational regime they experienced under Seksunthorn marked a decisive escalation in disciplinary intensity and ideological purpose. But it did not emerge from nowhere. The groundwork had already been laid. Under British-trained headmasters like Sutton, students had come to understand themselves as future leaders, bound by duty, discipline, and national significance. The school’s rituals, hierarchies, and alumni networks had already begun to cultivate the emotional infrastructure of fraternal loyalty. What the 1934–1941 cohort encountered was a redirection rather than a rupture: the soft paternalism of pre-revolutionary schooling was replaced with a harder, more militarized vision of the state. Yet the emotional architecture remained intact. It was this blend of ideological transformation layered atop existing structures of institutional belonging that explain how a cohort so deeply divided by Cold War violence could later coalesce around a shared ideal of national stability. They had been trained to interpret their experiences not only as personal, but as formative of national life.

. As the cohort aged, their memories, never inert, were mobilized in selective and strategic ways. Alumni reinterpreted earlier violence as the foundation of virtue, elevating Seksunthorn to the status of moral patriarch and recasting Suankularb as a crucible of ethical leadership. That they could do so reveals the malleability of institutional memory as well as the political utility of shared emotional inheritance.

By attending to these emotional, ideological, and institutional afterlives, I argue for a fuller reckoning with how elite schools shape political subjectivity. Suankularb was not merely a feeder school for civil servants or a site of curricular reform; it was a stage on which authority, hierarchy, and national purpose were rehearsed and felt. As Thai Studies continues to grapple with the legacies of authoritarianism and elite formation, such affective inheritances and the contradictory attachments they engender deserve sustained attention. These students may have passed through a school, but what they inherited, and later reassembled, was a vision of the state that felt personal and enduring.

## Footnotes

1 Thanin’s ministerial appointments were unpopular, contributing to the instability of his regime. Pinyo alienated his ministerial colleagues with his condescending demeanor. After Thanin’s downfall, they threatened to shoot him with fireworks should he attempt to retrieve his belongings (Montri 1978, 270).

2 Thanin 1994, 33.

3 *Cremation Volume of Air Marshal Sak Tharichat* 1994, 28.

4 Samreung 1982, 75.

5 Samreung 1982, 77.

6 SKRC 1995, 502, 277.

7 *School Thai* April 22, 1980 quoted in Lada 1982, 145.

8 McCargo 2005; 2021.

9 Murashima 2009, 23.

10 Somkhwam 1984, 179.

11 Murashima 2009, 24-26.

12 Sutachai 2015, 17-18; Charoen 2015, 112-113.

13 Charoen 2015, 131.

14 Vatthana and Ninlawadee 2021.

15 The group was later implicated in the assassination of eighteen members of the Farmer Federation of Thailand. See Prajak 2006, 20.

16 Puey 1977, 11.

17 Hyun 2023, 117.

18 Many people believe that more than one hundred were killed. Sudsai denied Red Gaur involvement in the killings, claiming in later decades that they had been scapegoated by authorities (Thongchai 2020, 191–195).

19 Somsak, 2022.

20 Morrell and Chai-anan 1981, 322.

21 Quoted in *Statesman Foundation* 2019, 50.

22 SKEC 1988, 260.

23 Chalonglak 1985, 273.

24 Sawat 1984,193-4.

25 Thanin 2009, 16.

26 Thanin 2009, 15.

27 Wyatt 1969, 35-62.

28 Damrong 1963, 27.

29 Bunchuea 1957, 16.

30 Chulalongkorn 1963, 29.

31 Damrong, 1946:203

32 Quoted in *SKRC* 1995, 444.

33 Wyatt 1969, 70-72.

34 Si Sunthorn, 1959, 80.

35 Phae 1959, 49. Damrong’s (1946, 201) reflections on the gentle discipline he received at Bowonniwet lend weight to scholarly suggestions that monastic punishment practices were stratified by class. While surviving documentation is limited (Cf. Wyatt 1969, 21; McDaniel 2008, 83), existing sources suggest that lower-status students were subjected to harsher treatment. Western accounts frequently noted the disordered nature of temple schooling (Cf. McFarland 1884, 210–212). Yet Phae Talalaksana reported routine corporal punishment in some monasteries. Offences like “avoiding everything by sleeping” or “escaping after waking” were punished with three, five, or ten strokes. In extreme cases, monks caned students while the students were bound and suspended to induce “great emotional distress” (Phae 1959, 51).

36 Laksamansuphot 1963, 5.

37 Bidyalongkorn 1963, 44.

38 Education Ministry Letters 1897-1899.

39 Kitiyakara 1898.

40 For full charter see SKRC, 1995:121-122.

41 Pia 1910.

42 Insri 1985, 89.

43 In 1885, Suankularb hired Reverend J. Wastie Green, an Oxford MA in world religions, to teach basic English. Students, struck by his resemblance to a “British-style monk” in his black cap and gown, were scandalized to discover he drank alcohol. Dubbing him “defrocked Green” (*sami khiao*), they speculating he had been excommunicated from his English temple and forced to teach children in the tropics. Green was succeeded by Mr. Thompson and an unnamed third teacher, both of whom departed within a year of their engagement (Bidyalongkorn 1963, 46).

44 Young 1898, 56.

45 Bartle 1976.

46 Insri 1985, 90.

47 Insri 1985, 90.

48 Insri 1985, 94. Truancy remained common well into the 1910s. Students frequently spent afternoons at nearby Wat Suan Pleum, where they amused themselves by blowing marijuana smoke at the temple’s blind crocodile, *Ai Bod*, or watching participants in *sak yant* ceremonies receive tattoos and enter trance-like states.

49 Chitra 1966, 24.

50 Quoted in Sawat 1959, 107.

51 Sawat 1959,113.

52 Sangop 1982, 100.

53 Samretwannakit 1960, 202.

54 Pradit 1986, 43.

55 Pathom 1957, 68.

56 SKRC 1995, 381. Four King’s Scholars studied at Borough Road: Suankularb alumus Chaophraya Thammasakmontri (1876-1943), a future education minister (1896); Phraya Methathibordee, future director-general of the Department of Curriculum and Instruction Development (1901); Major-General Suksasombun, who later served as headmaster of Suankularb Front Palace School (1903); and Phraya Phontracha, a former Suankularb teacher and later chancellor of Chulalongkorn University (1907).

57 *Suankularb Withaya* 1931a.

58 By 1910, a Suankularb graduate could enter the military academy, ascend to sub-lieutenant, and earn a comfortable monthly salary of eighty baht within two years (Insri, 1985, 92). Still more lucrative was the legal profession; after a stint of training at the Ministry of Justice, graduates could earn up to 240 baht per month, sixty times the monthly salary of a provincial schoolteacher.

59 SKRC 1995, 162.

60 Pradit 1986, 39.

61 Johnson converted to Buddhism, became fluent in Thai, and spent the rest of his life in Siam. As Chief Inspector, he standardized national language assessments and advised three successive ministers of public education, including Suankularb alumni Pia Malakul (1912–1916) and Chaophraya Thammasakmontri (1916–1926; 1932–1933). His son later recalled that King Chulalongkorn had considered Johnson for the post of minister of education, though Johnson declined, citing his foreign origins (Pathom 1957).

62 Sawasdisansataraput 1958, 20.

63 Whitehouse 2022, 99-103.

64 Chanchai et al. 2010

65 SKRC 1995, 163.

66 Sawat 1959:110.

67 *Bangkok Times*, quoted in Sawat 1959, 111-112.

68 SKRC 1995: 180; Insri 1985: 90; Bunyoptham 1956: 57

69 *Suankularb Withaya* 1927, 304. This reportedly was the first use of a fire engine in Siam.

70 *S*uankularb Withaya 1931b: 863

71 Bidyalongkhorn 1963, 45.

72 Prakep 1970, 125.

73 Prakep 1970, 136. Somjit Khosawana (quoted in SKRC 1995, 215) another student eyewitness, recalls that Sanguan, incensed by a student’s question, brandished his firearm threateningly and declared, “No questions. Any further enquiries will be met with force. The nobles are already in our custody.”

74 Pridi 1978, 64.

75 Stowe 1991,18.

76 *Bangkok Daily Mail* September 12, 1932.

77 Pipat, 1984, 95; SKRC 1995, 215; Prakep 1979, 137.

78 SKRC 1995, 215.

79 Pradap 2003, 34. Prem was close school friends with Prasong and promoted him to vice-admiral in 1981.

80 Surasak 2006, 33-57.

81 Chek 1984, 81.

82 The Bowowardet rebellion broke out on October 11, 1933 and lasted for two weeks. It was led by Prince Bowowardet (1877-1953), a royalist and opponent of the People’s Party. After the rebellion was crushed he fled to Cambodia, where he remained in exile until 1948.

83 Suksuan 1985, 239; Kamol 1984, 210.

84 Somkhwan 1984, 185.

85 Chinwut 1984, 89.

86 Chinwut 1984, 95.

87 Duang Yadam 1984, 70.

88 Somkhwan 1984, 181.

89 SKRC 1995, 233.

90 Churchill left Siam in 1938, followed soon after by Mr. Deagan, who returned to England shortly before the Second World War. Herbert Somerset Silverthorn, a respected mathematics and English teacher (and briefly tutor to Prince Bhumibol in 1934), remained at Suankularb until his internment during the Japanese occupation. After he was released at the end of the war he returned to London, where he committed suicide by jumping from Southwark Bridge on March 28, 1946. His death certificate stated: “The deceased did take his own life whilst the balance of his mind was disturbed.”

91 Barmé 1993, 129.

92 SKRC 1995, 228. Students recall that Mr. Si never let a hungry boy go unfed, and on match days, bought flower garlands to adorn the football players. When the school’s King’s Scholars embarked on their journeys to Europe, Mr. Si travelled to the docks to see them off, lingering by the water’s edge until their ship disappeared around the riverbend (S.K. 8146 1984, 89-90).

93 Kamol 1984, 210.

94 S.K. 8289 1985, 215.

95 Anukit 1984, 172.

96 SKRC 1995, 226.

97 The Raj-style jacket is a collarless white coat that was commonly worn as formal Thai attire in the early twentieth century. The *chong kraben* is a traditional lower-body garment which is wrapped around the waist with a fold drawn between the legs and secured at the back.

98 Samretwannakit 1960, 205; Sansanee 2004, 81. The habit killed him; he died of esophageal cancer in 1941, at the age of 46*.*

99 Wira 1985, 344.

100 Prakep 1970, 109.

101 For comparison, contemporaneous accounts from Edwardian Britian demonstrate that caning was commonly administered to hands, with six strokes considered a serious punishment (see Middleton, 2008, 264).

102 Choti 1984, 129.

103 Wira 1985,345.

104 Prakep 1970, 109-110.

105 Prakep 1970, 106.

106 Wira 1985, 344.

107 Duang Ya Dam 1984, 70-5. The school newspaper once satirized Seksunthorn’s dislike of students gazing out the window in a playful verse:
*Do not show your face to enjoy the view* *If you disobey, Luang Sek will fume* *He’ll walk briskly* *With a cane in hand*. *Whoever gets hit often groans, “Ooh, ooh..”* (quoted in Wilas 2005, 182).

108 Wira 1985, 345; Duang Yadam, 1984, 69-76.

109 Samretwannakit 1960, 209.

110 Pichit 1984, 338; SKRC 1995, 220.

111 S.K. 2010 1984, 155.

112 Wira 1985, 345.

113 Sawat 1984,193.

114 SKRC 1995, 443.

115 Pichit 1984, 338.

116 Prakep, 1970:110

117 Prakep 1970, 110.

118 Suksuan 1985, 266-267.

119 Suksuan 1985, 267.

120 Suthin 1985, 183. Anecdotal evidence suggests that similar regimes were established at other schools in Bangkok. At Thepsarin, Kumut Chandruang recalls being so frightened by the physical punishments dispensed by one teacher that he sought refuge in the city’s billiard halls, where he gambled away his dead mother’s jewelry (Kumut, 1940, 164-166)

121 Chinwut 1984, 90.

122 Duang Yamdam 1984, 70-75.

123 Anukit 1984, 172.

124 Samretwannakit 1960, 201.

125 Quoted in Samretwannakit 1960, 210.

126 Samretwannakit 1960, 201.

127 Samretwannakit 1960, 211.

128 Quoted in Samretwannakit 1960, 202.

129 Samretwannakit 1960, 210-211. When Churchill returned to England in 1938, Seksunthorn was briefly appointed headmaster.

130 Choti 1984, 117.

131 Samretwannakit 1960, 204.

132 Quoted in SKRC 1995, 444.

133 Anukit 1984, 172.

134 Ruam quoted in Murashima 2009, 16.

135 Somchai 1993, 142.

136 Somchai 1993, 142-144.

137 Prakep 1970, 111-114.

138 Prakep 1970, 113.

139 Prakep 1970, 113.

140 Wilas et al. 1960, 21-22.

141 Wilas et al. 1960, 23.

142 On Ruam Wongphan’s execution and Charoen’s rural mobilization, see above.

143 Kasian 2011.

144 Prajak 2006, 20.

145 Baker and Pasuk 2022, 260.

146 Sommay 23-25, 2011.

147 *Suankularb Student Committee* 1974.

148 Buddhist Theology and Culture Group of Suankularb Withayalai 1975, x.
